# 24 Scoliosis in children with juvenile idiopathic arthritis: any link?

**DOI:** 10.1093/rheumatology/keac496.020

**Published:** 2022-10-07

**Authors:** Ben Aissa Rania, Ferjani Hanene, Moalla Mariam, Ben Nessib Dorra, Triki Wafa, Maatallah Kaouther, Kaffel Dhia, Hamdi Wafa

**Affiliations:** Department of Rheumatology, Mohamed Kassab Institute of Orthopedics, Tunis, Tunisia

## Abstract

**Background:**

Juvenile idiopathic arthritis (JIA) is an unusual aetiology of scoliosis in childhood. With few data in literature, the associated factors of scoliosis in JIA are still unknown.

**Objectives:**

To describe the incidence and associated factors of scoliosis in children with JIA.

**Methods:**

A cross-sectional study including children with JIA according to the International League of Associations for Rheumatology (ILAR). The recorded data included anthropometric measurements (weight, size, body mass index (BMI), and leg length), disease characteristics (JIA subtypes and duration), and static foot posture disorders. Regarding scoliosis, we collected clinical and radiographic findings.

**Results:**

Thirty-five patients, forty-three percent of the patients were boys (*n* = 15). The mean age was 12.2 ± 3.61 years. The mean disease duration was 4.1 ± 3.29 years. The mean patient global assessment and the mean visual analogic scale were 3.4 ± 3.02 and 3.37 ± 2.92, respectively. The patient's distribution of JIA subtypes was oligoarticular (*n* = 13), enthesitis-related arthritis (*n* = 9), polyarticular (*n* = 4), undifferentiated (*n* = 4), psoriasis-related JIA (*n* = 4) and systemic-onset (*n* = 1). The mean CRP and ESR were 7.51 ± 11.85 mg/l and 18.88 ± 15.53 mm, respectively. The mean JADAS was 7.58 ± 6.3. Seventeen percent of the patients had an inactive disease (*n* = 6). Seven patients (20%) (6 females) had developed scoliosis during their disease, the spine regions of scoliosis were thoracolumbar scoliosis (*n* = 6) and thoracic scoliosis (*n* = 1). There was no specific distribution of scoliosis among JIA subtypes (*p* = 0.464). Most patients with scoliosis had associated leg length discrepancy (71.43% *vs* 28.57%, *p* = 0.025). Static foot posture disorders were present in most patients with scoliosis (85.71% *vs* 14.29%) but this difference was not significant (*p* = 0.673) compared with patients with no scoliosis. Weight, size and BMI were not associated with scoliosis (*p* = 0.955, *p* = 0.922, *p* = 0.303 respectively).

**Conclusion:**

Our study shows that scoliosis is associated with leg length discrepancy. Indeed, the joint inflammation of the lower limb may explain the postural syndrome in children with JIA, with a prompt impact on the axial skeleton.

